# A robot-aided visuomotor wrist training induces motor and proprioceptive learning that transfers to the untrained ipsilateral elbow

**DOI:** 10.1186/s12984-023-01258-w

**Published:** 2023-10-24

**Authors:** Huiying Zhu, Yizhao Wang, Naveen Elangovan, Leonardo Cappello, Giulio Sandini, Lorenzo Masia, Jürgen Konczak

**Affiliations:** 1 Human Sensorimotor Control Laboratory, School of Kinesiology, University of Minnesota, 1900 University Ave. SE, Minneapolis, MN 55455 USA; 2 Department of Rehabilitation Medicine, Tianjin Huanhu Hospital, Tianjin, China; 3 Department of Health and Exercise Science, Tianjin University of Sport, Tianjin, China; 4 The BioRobotics Institute, Scuola Superiore Sant’Anna, Pisa, Italy; 5 Department of Excellence in Robotics and AI, Pisa, Italy; 6 Department of Robotics, Brain and Cognitive Sciences, Istituto Italiano di Tecnologia, Genova, Italy; 7 Institut für Technische Informatik, Universität Heidelberg, Heidelberg, Germany

**Keywords:** Human, Learning, Motor, Proprioception, Robotic Rehabilitation, Somatosensory

## Abstract

**Background:**

Learning of a visuomotor task not only leads to changes in motor performance but also improves proprioceptive function of the trained joint/limb system. Such sensorimotor learning may show intra-joint transfer that is observable at a previously untrained degrees of freedom of the trained joint.

**Objective:**

Here, we examined if and to what extent such learning transfers to neighboring joints of the same limb and whether such transfer is observable in the motor as well as in the proprioceptive domain. Documenting such intra-limb transfer of sensorimotor learning holds promise for the neurorehabilitation of an impaired joint by training the neighboring joints.

**Methods:**

Using a robotic exoskeleton, 15 healthy young adults (18–35 years) underwent a visuomotor training that required them to make continuous, increasingly precise, small amplitude wrist movements. Wrist and elbow position sense just-noticeable‐difference (JND) thresholds and spatial movement accuracy error (MAE) at wrist and elbow in an untrained pointing task were assessed before and immediately after, as well as 24 h after training.

**Results:**

First, all participants showed evidence of proprioceptive and motor learning in both trained and untrained joints. The mean JND threshold decreased significantly by 30% in trained wrist (M: 1.26° to 0.88°) and by 35% in untrained elbow (M: 1.96° to 1.28°). Second, mean MAE in untrained pointing task reduced by 20% in trained wrist and the untrained elbow. Third, after 24 h the gains in proprioceptive learning persisted at both joints, while transferred motor learning gains had decayed to such extent that they were no longer significant at the group level.

**Conclusion:**

Our findings document that a one-time sensorimotor training induces rapid learning gains in proprioceptive acuity and untrained sensorimotor performance at the practiced joint. Importantly, these gains transfer almost fully to the neighboring, proximal joint/limb system.

## Introduction

Within the context of motor learning, transfer of learning refers to how an acquired skill can be executed in a new context, a new workspace, or how a learnt motor pattern transfers from one effector system to another [1–3]. Of specific interest has been to determine what parameters influence such learning and, importantly, whether it transfers to other motor systems, such as between homologous muscle systems such as those controlling the left and right hand. There is evidence of inter- and intralimb transfer of motor learning [1, 4–6]. That is to say, untrained limb systems exhibit signs of motor learning without practice. Moreover, neuromotor systems adapt to unknown force fields [7, 8] and the learning of such new dynamics studies induces observable changes in the movement kinematics and kinetics of the untrained limb [9].

While there is solid evidence for motor learning transfer, the transfer of proprioceptive or somatosensory learning has received less attention. This is noteworthy given the fact that proprioceptive signals are essential for motor learning and that the major neural somatosensory and motor cortical areas have substantial reciprocal connections [10]. Moreover, there is solid evidence that proprioceptive and motor learning is bidirectional [11–13]. That is to say, gains in motor performance are associated with concurrent gains in proprioceptive function such as an increase in position sense acuity and vice versa [14].

With respect to the transfer of proprioceptive learning, recent work from our group documented that a short 45-min visuomotor training of the right wrist significantly reduced position sense thresholds in the trained wrist and that these gains in proprioceptive function transferred to the contralateral left wrist [15]. Interestingly, the position sense acuity of both wrists improved at nearly the same rate as their respective JND thresholds were reduced by approximately 30% at the end of training. Importantly, the time scale of memory consolidation differed for the transfer of proprioceptive and motor learning. The gains in proprioceptive acuity were measurable immediately after training and decayed quickly within 24 h, while a motor transfer was only observed 24 h past testing. In that study the fast decay of proprioceptive learning was likely owed to the short training period. A previous report [11] employing a robot-aided wrist visuomotor training over 5 days documented that consolidation of proprioceptive learning was observed over several days with improvements in position sense acuity still measurable up to 5 days after the last training.

The current study was designed to enhance our knowledge about the magnitude and extent of ipsilateral transfer of motor and proprioceptive learning. The term ipsilateral here refers to the transfer of learning to adjacent joints within the same limb that are controlled by different, non-homologous muscles. Using a robotic wrist exoskeleton, healthy adults learnt a visuomotor task that required them to make increasingly precise, small amplitude wrist movements. We then determined position sense thresholds of the wrist and the adjacent elbow to obtain a psychophysical marker of proprioceptive learning. In addition, participants performed a goal-directed pointing task that they had not practiced before and after training to assess to what extent motor learning had transferred from the wrist/hand to the elbow/forearm motor systems. Finally, we examined how well such learning retained after 24 h.

## Materials and methods

### Participants

Fifteen right-handed healthy adults (age: 23.5 yrs. ± 4.9 yrs.; 5 males) were recruited for this study. All participants gave written informed consent prior to testing. The study protocol was reviewed and approved by the Institutional Review Board at the University of Minnesota’s Human Research Protection Program. All participants reported no health problems and no known neurological conditions at the time of testing. Handedness of the participants was determined by the Edinburgh Handedness Inventory [16].

### Devices

**Fig. 1:**
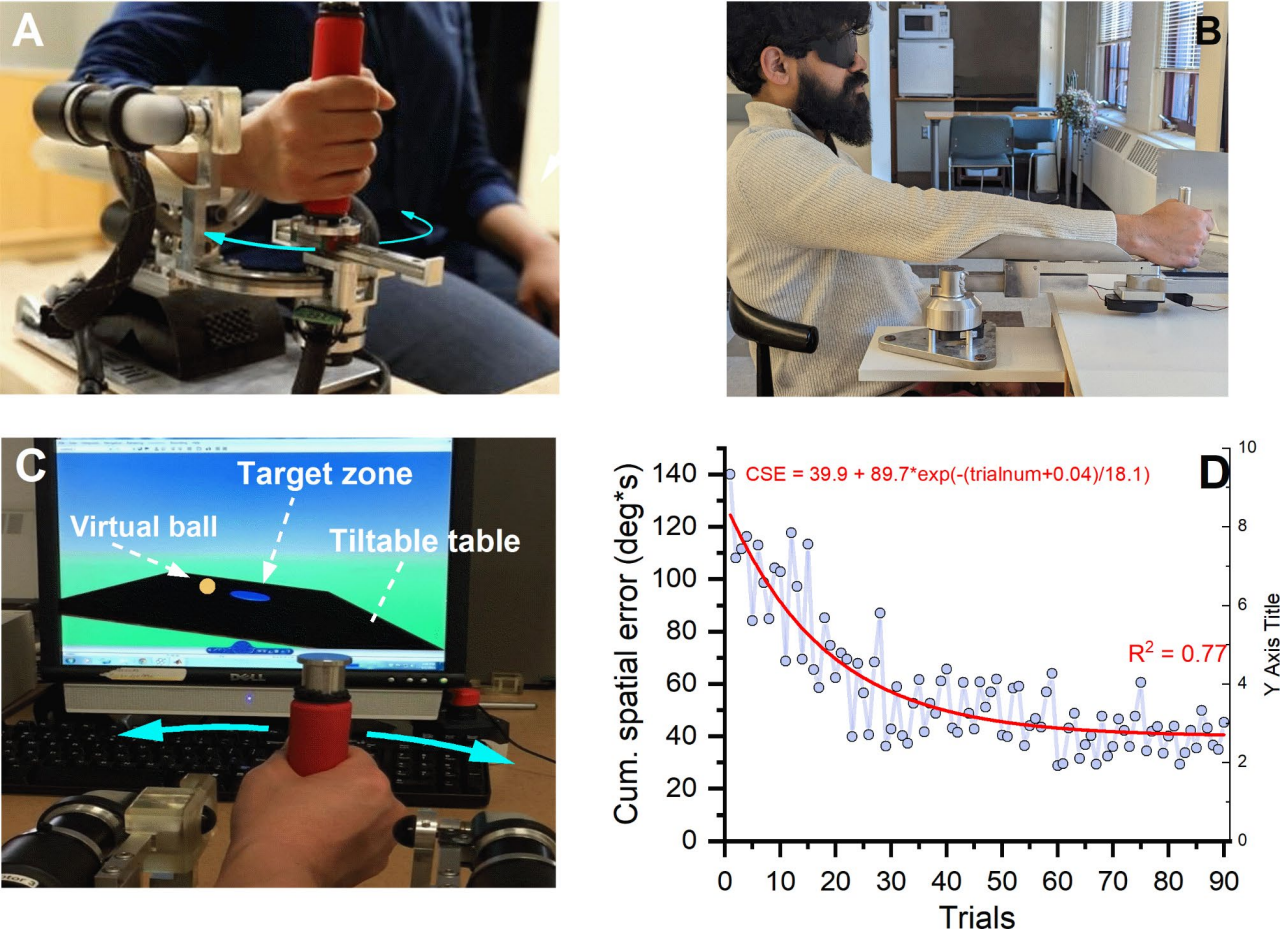
Experimental setup. (**A**) Frontal view of the 3 DOF robotic wrist exoskeleton. This study only required users to make wrist flexion/extension movements. (**B**) The elbow-joint manipulandum used for the transfer task. It allowed elbow flexion/extension movements in the horizontal plane. (**C**) Visual display as seen by the learner. Wrist flexion/extension movements tilted the virtual table. Learner attempted to roll the virtual ball into the target zone. (**D**) Learning effect on movement trajectory formation as measured by the Cumulative Spatial Error (CSE). Each data point represents the mean CSE of all participants for a particular trial. Note the decline of CSE over successive trials. Red line indicates the fit of the exponential decay function

*Wrist Robot:* A three degree-of-freedom robotic exoskeleton (Fig. 1A) was used for the evaluation of wrist proprioceptive and motor function at pre-, posttest and retention as well as for the training sessions. It allows for motion within the full range of movement of the human wrist/forearm around its degrees-of-freedom (i.e., wrist flexion/extension, wrist adduction/abduction, and forearm supination/pronation). The wrist robot is a fully backdrivable system, powered by four brushless motors with the capability of delivering precise position and velocity stimuli to the wrist. The robot accurately encodes the wrist position at 200 Hz with a spatial resolution of 0.0075°. In addition, the robot is integrated with a virtual reality environment that in this study displayed the training task and provided instantaneous visual feedback of the user’s wrist position during the training session.

*Elbow joint manipulandum*: A custom-built 2-joint manipulandum (see Fig. 1B) was used for assessing a possible transfer of proprioceptive and motor learning to the elbow. Technically, the manipulandum allows rotation around the wrist and elbow. Here, participants only performed elbow extension/flexion in the horizontal plane while the wrist joint movement was blocked during testing. A laser was attached to the front of the device to indicate the current arm position. Two US Digital H6 optical encoders (2500 quadrature count/revolution; spatial resolution: 0.036°), housed at the rotating point of the manipulandum lever arm segments, recorded angular position at a sampling frequency of 200 Hz.

### Experimental protocol

**Fig. 2:**
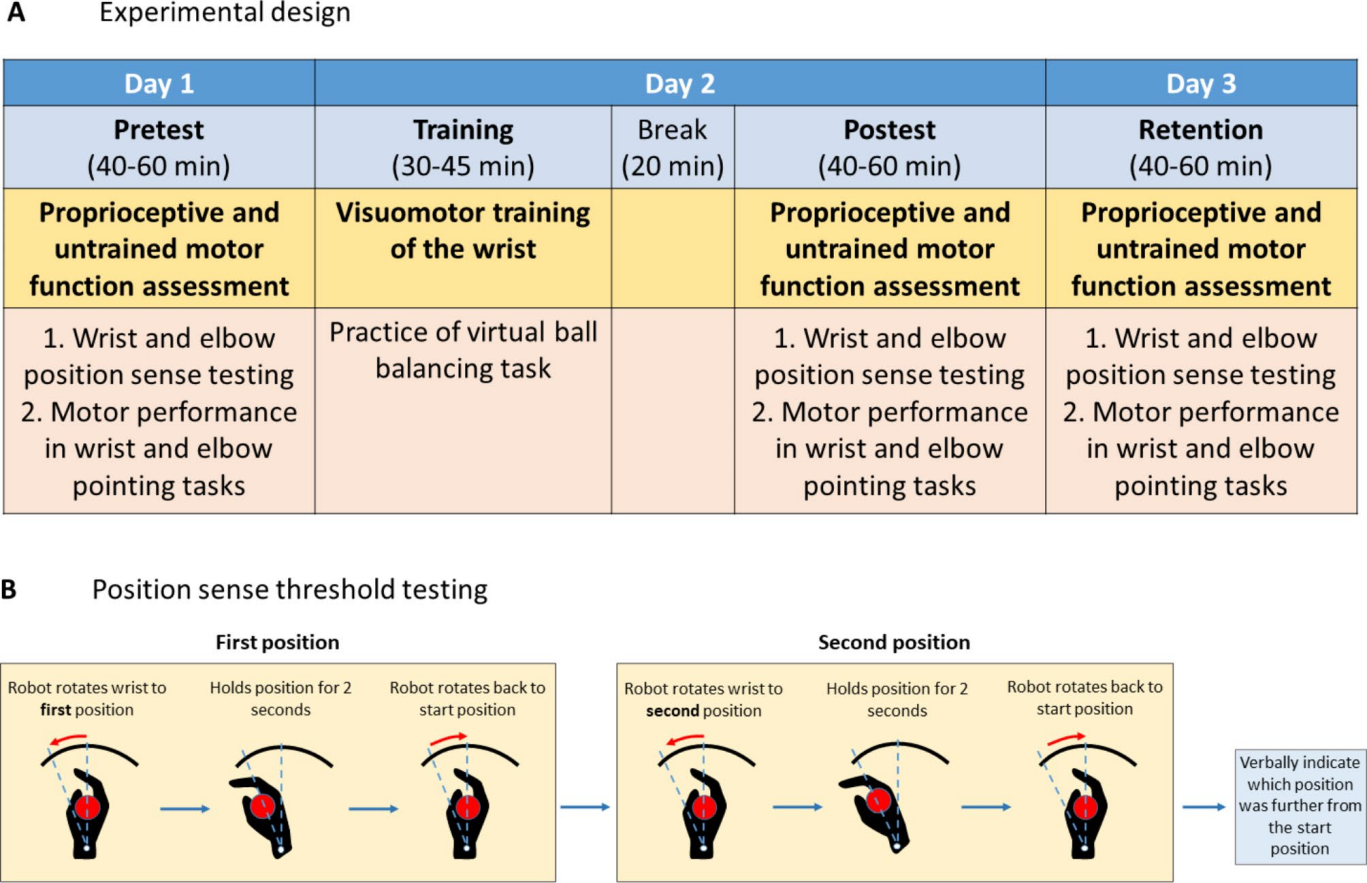
Schemata of experimental design and the procedure for position sense testing of the wrist. For assessing the elbow joint position sense, the procedure was analogous but used the elbow manipulandum (see Fig. 1B).

The protocol comprised 4 sessions spread over three days: At Day 1, participants’ proprioceptive acuity and motor performance in a goal-directed pointing task were assessed at both wrist and elbow (pretest). On Day 2, participants completed the visuomotor training and the proprioceptive acuity and motor performance assessments were repeated (posttest). A final retention assessment was completed 24 h later on Day 3 (Fig. 2A). Prior to the pretest on Day 1, all participants underwent practice trials to get familiar with the devices and the tasks. The order of assessments (proprioception or motor) was randomized between subjects to account for possible order effects.

The evaluation of proprioceptive acuity consisted of a psychophysical assessment of wrist and elbow position sense using a two-alternative forced-choice discrimination paradigm. In each trial, the device passively moved the joint (right wrist or right elbow) at a constant velocity of 6°/s from the neutral position to either a *standard* (15° for the wrist, and 20° for the elbow) or a *comparison* position (> *standard*). Participants were then asked to verbally indicate which of the two positions was more flexed (see Fig. 2B). The subsequent comparison stimulus for the next trial was then determined by size difference between the two experienced stimuli and the previous verbal response using an adaptive Quest algorithm [17]. During testing participants wore opaque glasses to block vision and wore headphones that played low-volume pink noise to mask any auditory cues. The validity and reliability of this robot-based proprioceptive assessment have been established previously [18].

To evaluate untrained sensorimotor performance, participants performed goal-directed pointing movements matching with right wrist and right elbow in an ipsilateral matching task. Like during proprioceptive testing, vision and audition was blocked. In each trial, the respective joint (wrist or elbow) was passively rotated from the neutral joint position to a target position (15° wrist flexion, and 20° elbow flexion), held for 2 s and then moved back to the neutral position. Subsequently, participants actively moved their forearm or wrist to the previously perceived target position. Each assessment consisted of 20 trials.

The protocol for the visuomotor training was identical to and described in detail in our previous paper on contralateral transfer [15]. In brief, participants watched a visual display and had to move a virtual ball rolling on a tiltable board into a target zone by making continuous wrist flexion/extension movements (see Fig. 1C). A trial was considered to be completed upon holding the ball within the target zone for 5 s. Consecutively, a new target zone was presented to begin the next trial. If the trial was completed within 60 s, it was considered successful. Between successful trials, the wrist position corresponding to the horizontal position of the table (where the ball would be stationary) was altered to either 10°, 15° or 20° of wrist flexion (relative to the neutral joint position). This allowed for the training of several distinct wrist flexion positions within the available range of motion of the joint. It promoted sensory-based learning across workspace of the joint. Participants were not informed of these changes in the balancing position. After a participant completed at least one successful trial in each of the three different wrist positions, the task difficulty was automatically increased by altering the following virtual mechanical properties: (1) increasing the virtual mass of the ball and increasing the gain of the velocity of the virtual ball, and (2) decreasing the friction coefficients on the virtual table. Participants used a movement range of 10° wrist extension to 40° wrist flexion to complete the training trials. To prevent fatigue, the training session was limited to a maximum of 90 training trials or 45 min. At optimal performance (every trial was successful) a participant would have completed 30 levels of difficulty. Participants were allowed a 2-minute break after every 30 trials.

### Measurements and statistical analysis

#### Evaluation of task-specific motor learning

To evaluate the effects of motor learning during the trained visuomotor task, the wrist angular time-series data of all participants were recorded during training using the signals of the position encoders of the robot. Instantaneous lateral deviation (LD) of the current wrist position relative to the neutral wrist position required to balance the ball was computed. In addition, Cumulative Spatial Error (CSE) for each trial was calculated using the equation below:

$$CS{E_{trial}} = \int_{i = 1}^n {\sum | } L{D_i}|dt$$

Where *n* is the last sample of each trial and trial onset (i = 1) is defined as the appearance of a new target zone. In addition, movement time (MT) of each trial was determined as the time difference between the appearance of a new target zone and the time when the virtual ball was held in the target zone for 5 s by the participant. Changes in CSE and MT represent measures of task-specific motor learning. Given that the trained virtual ball balancing task required learners to make continuous corrective movements until the ball was in the target zone, CSE reflects movements in both directions (flexion and extension).

#### Evaluation of proprioceptive acuity

Proprioceptive acuity was evaluated in the wrist position sense discrimination task described above. Participants’ verbal responses and the corresponding *stimulus difference size* (angular difference between comparison and standard positions) were recorded after every trial. Based on the verbal responses, a *Just-Noticeable Difference* (JND) threshold was determined by fitting the correct response rate and the stimulus difference size using a logistic Weibull function [19]. The JND or the marginal threshold x slope posterior distribution was derived by summating across the lapse rate dimension using the following psychometric function:

$${p}_{\alpha ,\beta }\left(\alpha =a, \beta =b\right)=\sum _{l}p(\alpha =a, \beta =b,\lambda =l)$$

where p(α = a, β = b, λ = l) is the full posterior distribution defined across the threshold values a, slope values b, and lapse rate l values that are contained within the parameter matrix defined a priori. The resulting JND threshold represented a measure of proprioceptive acuity.

#### Evaluation of untrained sensorimotor performance

Untrained sensorimotor performance was assessed by a discrete wrist or elbow pointing task described above. In each trial the participant’s joint was rotated to a target position. Subsequently, the participant actively rotated the hand or forearm to the previously experienced joint position. For each trial, the absolute angular error between the target position and the final joint position at the end of the active pointing movement was computed for each trial. A *Movement Accuracy Error* (MAE) was calculated as the absolute angular error between the target position and matching position across all trials for each participant using the equation below:

$$Movement\,Accuracy\,Error\,({\rm{MAE}})\, = \,\frac{{\sum\nolimits_{i = 1}^{20} {(|matching\,position - target\,position|)} }}{{20}}$$

### Statistical analysis

Distributions for all variables were examined for normality using the Shapiro-Wilk test. Outliers were defined as data points falling 1.5 times above or below the interquartile range (IQR) (20). One outlier was identified at wrist MAE measures during the retention tests and was removed from further analysis. The remaining values for both wrist and elbow datasets were normally distributed, and parametric statistical analysis procedures were employed. To determine immediate training related differences in proprioceptive acuity and motor performance, paired t-tests were performed on the outcome measures JND and MAE for the right wrist and elbow at posttest relative to pretest. For the same measures, paired t-tests were performed at retention (24 h after practice) relative to pretest to examine if possible training effects were retained. The performed t-tests were one-tailed test as previous work (15) had already established that JND and MAE decrease as a function of training and would not induce a deterioration of proprioceptive acuity. The initial significance level was set at p = 0.05. To account for multiple testing, false discovery rate corrections using the Benjamini–Hochberg procedure were applied (21). All statistical comparisons were performed by using the Statistical Package for Social Sciences (SPSS) version 24.0. Effect size (Cohen’s d) and power calculations were computed using G*power 3.1.

## Results

### Evidence of task-specific motor learning during visuomotor training

The majority of participants (14/15) finished the 90 training trials in the allotted time of 45 min. One participant completed only 60 trials within this time period. To document the extent of motor learning achieved during training, we computed the cumulative spatial error of the wrist joint trajectory for each trial. Mean CSE for the first ten trials across all participants was computed as 106.3 deg*s (SD: 15.3 deg*s), while the respective mean CSE for the last ten trials was 41.4 deg*s (mean SD: 7.4 deg*s) documenting that with practice participants tended to exhibit reduced lateral excursions (see Fig. 1D). In addition, movement time decreased with training with the mean MT_1–10th trial_ computed as 22.0 s (SD: 3.8 s) and the respective mean MT_final 10 trials_ as 17.8 s (mean SD: 3.0 s). The decrease in both movement parameters indicates that participants as a group completed the trials faster and with fewer spatial errors as they progressed through the training.

### Effects on position sense acuity and movement accuracy in the trained wrist

**Fig. 3:**
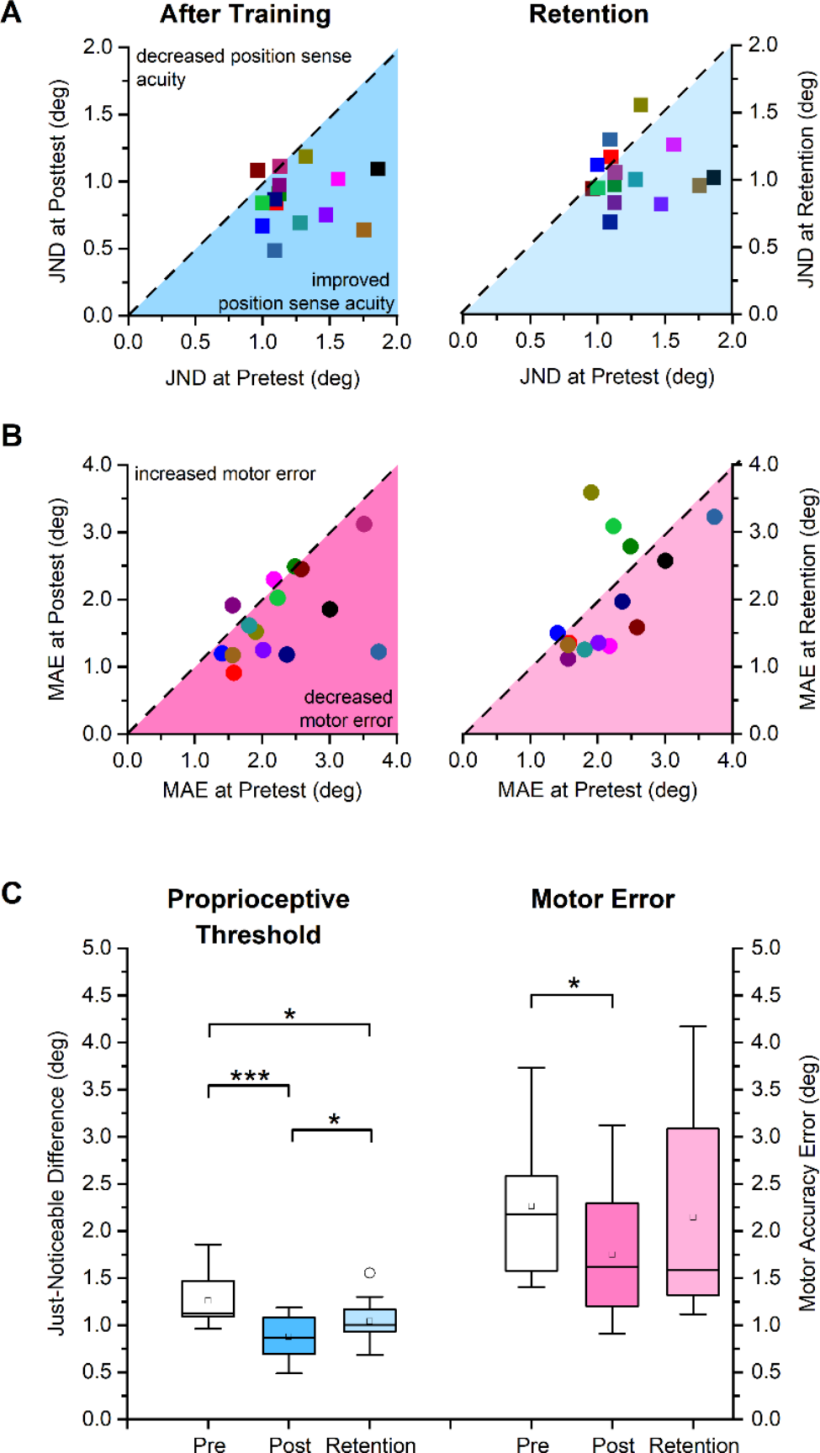
Sensorimotor effects of training on the trained right wrist as measured by the proprioceptive Just-Noticeable Difference threshold and the Movement Accuracy Error. (**A**) JND thresholds for each participant at posttest and retention are mapped against their pretest thresholds. Dashed diagonal line represents the line of equality, i.e., indicating that no change had occurred due to training. (**B**) Individual MAE values at posttest and retention are mapped against their pretest values. (**C**) Boxplot indicating the distribution of JND and MAE at pretest, posttest and retention. Boxes represent the range between 25th to 75th percentiles. Line within the box represents the median. The upper and lower whiskers extend to + 1.5 and − 1.5 inter-quartile range, respectively. Open circle symbol indicates an outlier value ( > ± 1.5 IQR). ***indicates p < 0.001, * indicates p < 0.05

In order to understand the effects of the visuomotor training task on the trained right wrist, JND and MAE of all the participants at posttest were compared relative to pretest. Participants exhibited a lower JND threshold after training (JND range at pretest: 0.96° – 1.86°; at posttest: 0.49° – 1.12°; see Fig. 3A). Mean JND decreased from 1.26° (SD: 0.28°) to 0.88° (SD: 0.20°). The corresponding mean relative change in JND between pretest and posttest was significant yielding a large effect size (30% decrease, *t* = 4.56, *p* < 0.001, *d* = 1.23, see Fig. 3C). With respect to the performance in the untrained wrist-pointing task, 13 of the 15 participants (87%) showed gains in movement accuracy (see Fig. 3B). Mean MAE was reduced from 2.22° (SD: 0.72°) to 1.77° (SD: 0.62°) at posttest, which corresponded to a significant mean relative change (20% decrease, *t* = 3.14, *p* = 0.0072, *d* = 0.74, see Fig. 3C).

To determine possible retention effects of training on the right wrist, JND and MAE of all participants at retention were compared. With respect to pretest, 11/15 participants (73%) retained the gains in proprioceptive acuity and 10/15 participants (67%) retained the gains in movement accuracy after 24 h (see Fig. 3A, B). A paired t-test between JND at posttest (mean: 0.88° ± 0.20°) and retention (mean: 1.04° ± 0.22°) revealed a significant difference in JND (*t* = − 2.17, *p* = 0.048, *d* = 0.59) indicating that proprioceptive acuity had somewhat decayed. However, when comparing retention relative to pretest, mean JND threshold was still significantly lower at retention (17% decrease, *t* = 2.65, *p* = 0.019, *d* = 0.65) showing that the effect of visuomotor practice was still present in the proprioceptive domain.

The respective comparison for MAE at posttest (mean: 1.77° ± 0.62°) and retention (mean: 2.18° ± 1.10°) failed to reach significance (*t* = − 1.40, *p* = 0.212, *d* = 0.38). Comparing motor performance at retention relative to pretest revealed that mean MAE at retention was not significantly different from MAE at pretest (2% decrease, *t* = 1.31, *p* = 0.212, *d* = 0.16) (see Fig. 3C). In summary, these results indicate that right-wrist visuomotor training enhanced its position sense acuity and movement accuracy in the untrained sensorimotor task. The improvement in position sense acuity was retained for up to 24 h, but performance gains in the untrained sensorimotor task were no longer significantly reduced at the group level.

### Proprioceptive and motor transfer effects in the untrained elbow

**Fig. 4:**
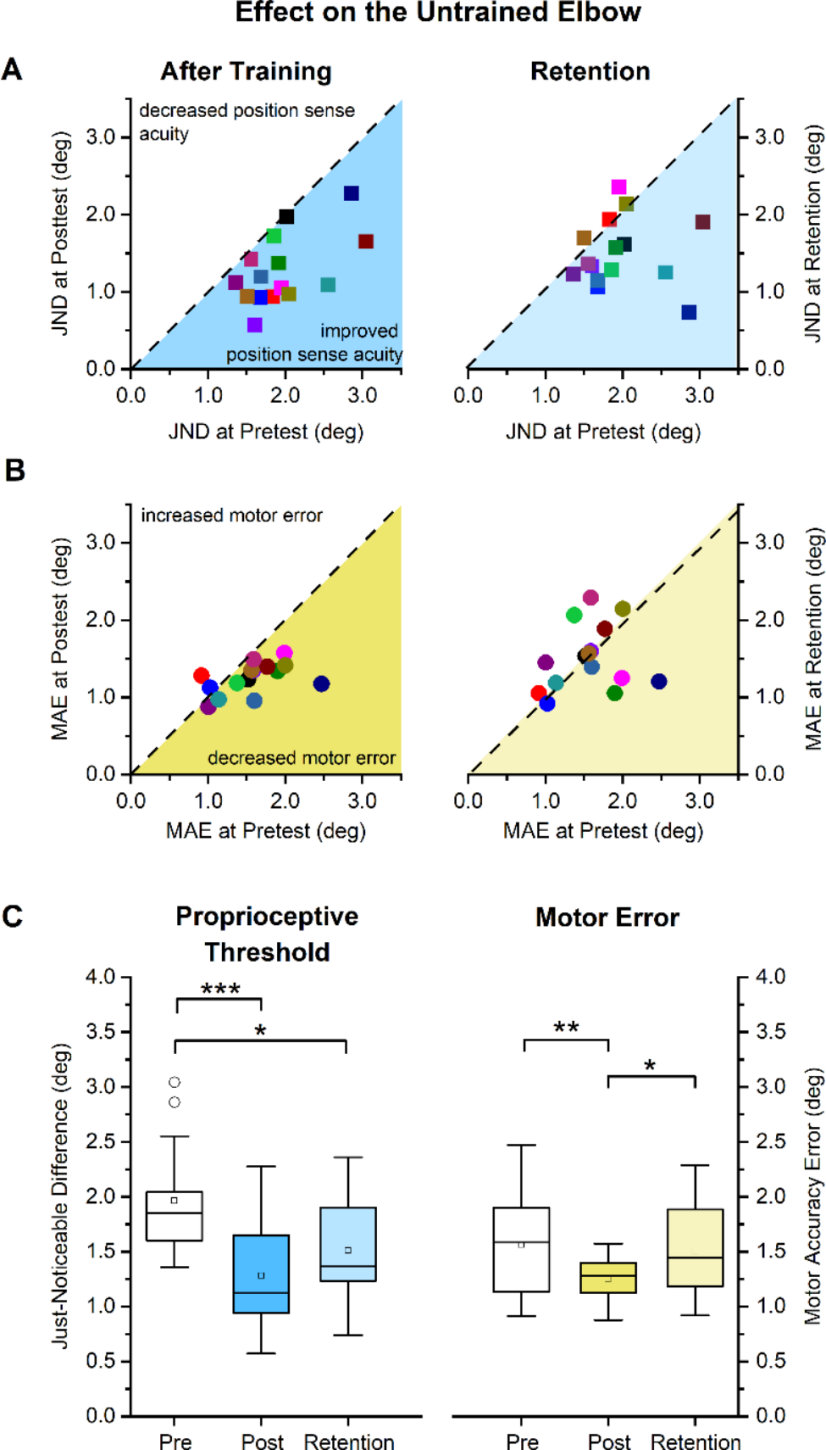
Sensorimotor effects of training on the contralateral untrained ipsilateral elbow as measured by the proprioceptive Just-Noticeable Difference threshold and the Movement Accuracy Error. (**A**) JND thresholds for each participant at posttest and retention are mapped against their pretest thresholds. Dashed diagonal line represents the line of equality, i.e., indicating that no change had occurred due to training. (**B**) Individual MAE values at posttest and retention are mapped against their pretest values. (**C**) Boxplot indicating the distribution of JND and MAE at pretest, posttest and retention. Boxes represent the range between 25th to 75th percentiles. Line within the box represents the median. The upper and lower whiskers extend to + 1.5 and − 1.5 inter-quartile range, respectively. Open circle symbol indicates an outlier value ( > ± 1.5 IQR). ***indicates p < 0.001, **indicates p < 0.01, *indicates p < 0.05

In order to determine the possible transfer effects of proprioceptive training in the untrained elbow, JND and MAE for the untrained elbow at posttest were compared relative to pretest. These data are shown in Fig. 4A and B (left panels). The subsequent paired t-test and effect size analysis revealed that JND was significantly reduced from 1.96° (SD: 0.49°) to 1.28° (SD:0.45°), which constituted a 35% decrease of JND at posttest (*t* = 5.83, *p* < 0.001) resulting in a very high effect size (*d* = 1.37, power = 1). The corresponding mean MAE data for the untrained the elbow-pointing task decreased by 20% from 1.56° (SD: 0.43°) to 1.25° (SD: 0.20°), a statistically significant difference (*t* = 3.22, *p* = 0.006, *d* = 0.83, power = 0.99; see Fig. 4C).

With respect to proprioceptive and motor performance at retention, the relevant within-subject pretest JND and MAE data are graphed against their retention values (see right panels in Fig. 4A, B). The mean elbow JND at retention had increased to 1.51° (SD: 0.85°) relative to posttest, but this difference was not statistically significant (*t* = 1.28, *p* = 0.204, *d* = 0.32, power = 0.39; see right panel Fig. 4C). Comparing mean JND between pretest and retention revealed that this difference was significant (23% decrease, *t* = 2.77, *p* = 0.015, *d* = 0.76, power = 0.98; see right panel Fig. 4C) indicating proprioceptive acuity had not decreased to the pretest level after 24 h.

Motor performance of the elbow-pointing task at retention revealed a significant rise in MAE from 1.25° at posttest to 1.51° (SD: 0.42°) at retention (21% increase, *t* = -2.51, *p* = 0.025, *d* = 0.65, power = 0.93; see left panel Fig. 4C), indicating that the training-related gains in untrained sensorimotor performance had significantly decayed 24 h after practice.

## Discussion

The main purpose of the study was to investigate whether proprioceptive and motor learning at one joint transfers to the adjacent joint of the same limb system. Specifically, we investigated whether practicing a task that only involved wrist movement would induce any observable changes in motor performance and proprioceptive acuity at the neighboring elbow joint. The major findings of the study are as follows: First, we found clear evidence of motor and somatosensory learning at the untrained elbow as the result of wrist practice. Movement accuracy in the previously not-practiced elbow-pointing task and elbow position sense acuity both showed significant gains of approximately 30%. Second, at the 24-hour retention assessment, gains in position sense acuity had consolidated, while gains in movement accuracy had significantly decayed. To our knowledge, this is the first study that documented ipsilateral transfer of learning in the motor and somatosensory domain.

### Concurrent sensory and motor learning

There is solid empirical evidence documenting that motor practice not only leads to improved motor function but can also induce somatosensory and visual perceptual change [13]. Such changes have been observed in the context of adaptive learning such as force-field adaptation, where learners have to adapt arm movement to unknown dynamics [22], or visuomotor adaptations [23]. These changes are also seen in visuomotor skill training. This study and previous work that applied a similar robot-based wrist movement training documented enhanced proprioceptive acuity in the trained wrist joint system and showed that such learning can extend to the untrained sensorimotor domain [14, 15].

What is remarkable is that these changes occur quickly. They do not require extensive practice over days but are observable after short bouts of practice. In this study learners did not practice for more than 45 min. The underlying processes of short-term plasticity in the somatosensory cortex associated with such learning are not fully understood. However, a neural correlate of such learning can be seen in median nerve somatosensory evoked potentials as increased P22-N24 amplitudes after training [24].

### Learning transfers to the ipsilateral elbow

The results of this study provide clear evidence of transfer in the sensory as well as motor domain between adjacent joints and limb segments. When comparing the learning gains achieved at the trained wrist with those of the proximal elbow joint, one observes, on average, a complete transfer of wrist-based proprioceptive learning to the ipsilateral elbow. As a group, participants reduced their JND thresholds by 30% at the trained wrist while the mean reduction at the elbow was 35% (see Figs. 3C and 4C). The corresponding mean reduction of movement accuracy error was 20% at both wrist and elbow indicating that motor transfer to an untrained movement was somewhat smaller in magnitude than the proprioceptive transfer.

The gains observed in this study are consistent with previous research reporting gains in proprioceptive acuity and motor accuracy (+ 30% and + 20%) after a similar robot-aided proprioceptive training of the wrist [14, 25]. This study complements earlier research on the transfer of somatosensory and motor learning to homologous joints in the contralateral body hemisphere, which showed learning gains that are very comparable in magnitude to those reported here [15]. That is, a training at a particular joint (here the distal wrist) induces sensory and motor gains of similar magnitudes in the adjacent joint as well as in the homologous, contralateral joint or limb system.

The neural mechanism underlying the observed ipsilateral transfer of proprioceptive and motor learning is not fully elucidated. However, it is well known that neural representations of elbow and wrist are located closely to each other and partially overlap in the somatosensory and motor cortical areas [26, 27], Moreover, the same motor cortical neurons may respond to loads experienced at the shoulder and the elbow during reaching [28]. It has been suggested that a certain amount of overlap in cortical representations aids the formation and control of functional muscle synergies [29]. Thus, it is plausible that ipsilateral transfer of proprioceptive and motor learning relies on neighboring or overlapping neuronal networks within the same cortical hemisphere, while the contralateral transfer is based on the activation of homologous brain areas in the opposite brain hemisphere [15].

### Retention of proprioceptive learning

In this study, learners underwent only a short, single training session of 30–40 min that led to substantial learning gains in proprioceptive and motor accuracy. What we show here is that these gains are remarkable stable and can still be observed after 24 h, which implies that some form of memory consolidation took place. Retention of proprioceptive learning was most pronounced at the trained wrist, but also present at the elbow in a subset of learners (see Fig. 4A C). Proprioceptive gains observed in this study are training related, as previous research established that repeated proprioceptive assessment testing with no training had little impact on the measures of proprioceptive acuity. Outcome measures of a second or third test were not inflated with respect to the first examination as shown by reliability coefficients between repeated measurements were over 0.97 [18]. In another study that applied the same procedures as used in the current study, a double baseline assessment of proprioceptive and motor function were performed [25] that yielded no differences between baselines. Additionally, the participants did not receive any feedback during testing and no knowledge of results was provided to the participant for any learning/adaptation to occur. Hence, the gains and its retention observed in proprioception can be attributed to training. In contrast, the gains in movement accuracy were no longer significant at retention testing for either joint. Based on this and similar research, empirical evidence suggests that motor memory consolidation either takes longer [15] than somatosensory memory formation, or the degree of spatial movement accuracy is not as great as observed in the proprioceptive domain [14] when considering short-term sensorimotor changes due to practice.

## Conclusion

This study provides empirical evidence that that a one-time sensorimotor training induces rapid learning gains in proprioceptive acuity and untrained sensorimotor performance at the practiced joint. Importantly, these gains may transfer fully to the neighboring, proximal joint. It complements earlier work from our group showing that a similar transfer is observable at contralateral, homologous joints and the respective limb system. That is, a picture emerges that sensorimotor practice induces measurable changes in proprioceptive and motor performance that extend beyond the trained limb system to those sensorimotor systems that are associated with overlapping or contralateral homologous representations in the somatosensory and motor cortices in both brain hemispheres. These results provide a scientific basis and hold promise for developing training programs for skill and neurorehabilitation training.

## Data Availability

The datasets of the current study are available from the corresponding author upon reasonable request. The dataset supporting the conclusions of this article is available in the University of Minnesota Data Repository: https://conservancy.umn.edu/handle/11299/166578.

## Abbreviations

CSE: Cumulative spatial error
DOF: Degrees-of-freedom
JND: Just-noticeable‐difference threshold
MAE: Spatial movement accuracy error
